# Supplementary material for: Oxytocin modulates local topography of human functional connectome in healthy men at rest
Source: Commun Biol. 2021 Jan 15;4:68. doi: 10.1038/s42003-020-01610-z (PMC7811009; doi:10.1038/s42003-020-01610-z)
Supplement: Supplementary file 2 — Supplementary Information [file 42003_2020_1610_MOESM2_ESM.pdf]

Oxytocin modulates local topography of human functional connectome in healthy men at rest

Daniel Martins, Ottavia Dipasquale, Yannis Paloyelis

Supplementary material

Supplementary Figure S1 – Effects of exogenous oxytocin on mean brain-wide functional connectivity. To examine whether exogenous oxytocin modulates mean functional connectivity of our brain-wide network, we first calculated mean functional connectivity as the average of the lower-triangle elements of the unthresholded connectivity matrix for each participant and treatment session. Then, we compared mean functional connectivity between our four treatment conditions using repeated measures one-way analysis of variance. In the upper panel, we present box and violin plots depicting the distributions of mean brain-wide functional connectivity for each treatment groups; middle horizontal lines represent the mean; boxes indicate the 25th and 75th percentiles (n=16 per treatment condition). In the lower panel, we present the descriptive statistics for each treatment condition.

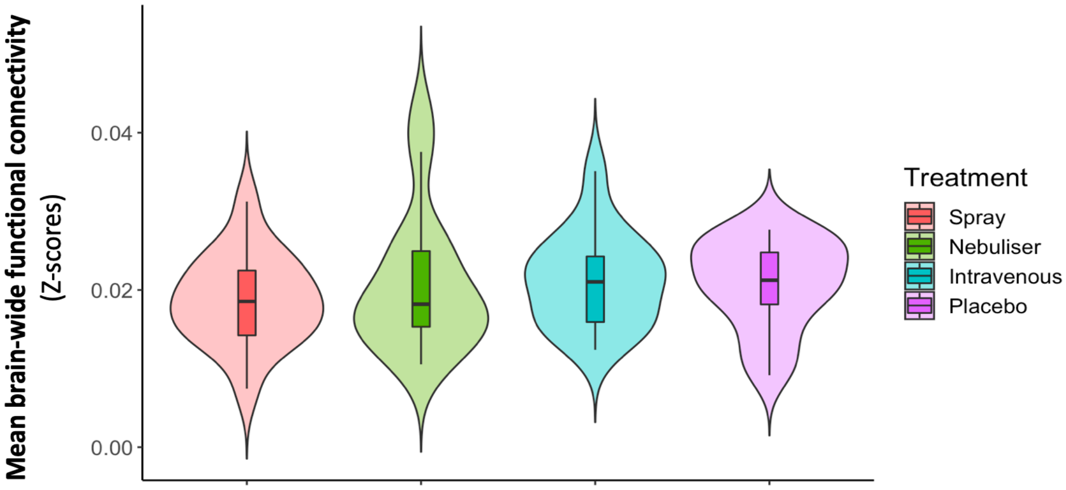

Treatment	Mean Functional Connectivity (Z-scores)	Standard error of the mean
**Spray**	0.0187	1.434×10^{-3}
**Nebuliser**	0.0216	2.640×10^{-3}
**Intravenous**	0.0214	1.500×10^{-3}
**Placebo**	0.0209	2.392×10^{-3}

Supplementary Table S1 - Effects of exogenous oxytocin on nodal betweenness-centrality. For each node, we first compared betweenness-centrality between our four treatment conditions using repeated measures one-way analysis of variance. Since we performed tests on multiple nodes, we controlled false positives using FDR correction for the number of nodes examined. When a significant effect was found, we explored this effect further using paired t-tests for each pair of our four treatment conditions, correcting for multiple testing using FDR. We present the results of this post-hoc investigation in this table. N. S. – not significant, p>0.05, after FDR correction for the number of post-hoc tests performed. We report the p-values after FDR correction.

Node	F	Post-hoc	Spray vs Placebo	Spray vs Intravenous	Intravenous vs Placebo	Nebuliser vs Placebo	Nebuliser vs Intravenous	Nebuliser vs Spray

	P FDR-corrected							
aMTG l (Middle Temporal Gyrus, anterior division Left)	9.974<br>0.036	T<br>P FDR-corrected	-2.627<br>0.019	-0.663<br>N.S.	-1.384<br>N.S.	-0.0971<br>N.S.	1.118<br>N.S.	1.736<br>N.S.
pMTG r (Middle Temporal Gyrus, posterior division Right)	10.333<br>0.013	T<br>P FDR-corrected	-3.198<br>0.005	-0.774<br>N.S.	-1.654<br>N.S.	-0.299<br>N.S.	1.321<br>N.S.	2.493<br>0.025
toMTG l (Middle Temporal Gyrus, temporo-occipital part Left)	8.632<br>0.046	T<br>P FDR-corrected	1.449<br>N.S.	1.034<br>N.S.	0.679<br>N.S.	2.409<br>0.029	2.214<br>0.042	0.618<br>N.S.
PostCG l (Postcentral Gyrus Left)	8.655<br>0.048	T<br>P FDR-corrected	1.682<br>N.S.	0.652<br>N.S.	2.159<br>0.047	1.094<br>N.S.	- 0.138<br>N.S.	- 0.669<br>N.S.
aSMG r (Supramarginal Gyrus, anterior division Right)	9.732<br>0.037	T<br>P FDR-corrected	1.018<br>N.S.	-0.111<br>N.S.	2.396<br>0.030	0.970<br>N.S.	0.644<br>N.S.	0.395<br>N.S.
pSMG r (Supramarginal Gyrus, posterior division Right)	10.028<br>0.023	T<br>P FDR-corrected	-3.023<br>0.009	-0.136<br>N.S.	-1.601<br>N.S.	-0.578<br>N.S.	1.179<br>N.S.	1.386<br>N.S.
iLOC l (Lateral Occipital Cortex, inferior division Left)	9.986<br>0.035	T<br>P FDR-corrected	2.454<br>0.027	2.713<br>0.016	0.634<br>N.S.	1.882<br>N.S.	1.336<br>N.S.	-1.246<br>N.S.
Precuneus	8.678<br>0.043	T<br>P FDR-corrected	-2.157<br>0.048	0.365<br>N.S.	-1.848<br>N.S.	-2.035<br>N.S.	0.132<br>N.S.	-0.320<br>N.S.
FOrb r (Frontal Orbital Cortex Right)	8.731<br>0.046	T<br>P FDR-corrected	2.227<br>0.042	0.600<br>N.S.	1.092<br>N.S.	0.481<br>N.S.	-1.382<br>N.S.	-1.612<br>N.S.
aPaHC r (Parahippocampal Gyrus, anterior division Right)	9.955<br>0.039	T<br>P FDR-corrected	0.456<br>N.S.	2.268<br>0.038	-2.574<br>0.021	-1.044<br>N.S.	0.716<br>N.S.	-1.757<br>N.S.
TOFusC r (Temporal Occipital Fusiform Cortex Right)	8.599<br>0.044	T<br>P FDR-corrected	2.134<br>0.049	1.901<br>N.S.	0.759<br>N.S.	1.731<br>N.S.	0.571<br>N.S.	-1.327<br>N.S.
OP r (Occipital Pole Right)	11.331<br>0.009	T<br>P FDR-corrected	1.259<br>N.S.	-1.759<br>N.S.	4.396<br>0.001	1.321<br>N.S.	-2.715<br>0.016	-0.412<br>N.S.
OP l (Occipital Pole Left)	9.924<br>0.031	T<br>P FDR-corrected	2.678<br>0.017	0.380<br>N.S.	2.636<br>0.019	1.567<br>N.S.	-0.239<br>N.S.	-0.627<br>N.S.
Amg r (Amygdala right)	9.937<br>0.033	T<br>P FDR-corrected	-1.549<br>N.S.	1.752<br>N.S.	-2.833<br>0.013	-0.126<br>N.S.	1.695<br>N.S.	0.922<br>N.S.
Cereb4-5 l (Cerebellum 4-5 Left)	9.962<br>0.035	T<br>P FDR-corrected	1.757<br>N.S.	-0.308<br>N.S.	2.408<br>0.029	1.514<br>N.S.	-0.102<br>N.S.	0.196<br>N.S.
Cereb7 l (Cerebellum 7b Left)	10.031<br>0.022	T<br>P FDR-corrected	0.250<br>N.S.	-0.521<br>N.S.	0.956<br>N.S.	2.749<br>0.015	0.889<br>N.S.	1.541<br>N.S.
Cereb9 r (Cerebellum 9 Right)	8.653<br>0.048	T<br>P FDR-corrected	-2.205<br>0.043	-1.659<br>N.S.	0.049<br>N.S.	-0.276<br>N.S.	-0.356<br>N.S.	1.899<br>N.S.
Cereb10 r (Cerebellum 10 Right)	10.341<br>0.012	T<br>P FDR-corrected	1.609<br>N.S.	-0.768<br>N.S.	2.975<br>0.009	1.859<br>N.S.	-0.769<br>N.S.	0.144<br>N.S.

Supplementary Table S2 - Effects of exogenous oxytocin on nodal local efficiency. For each node, we first compared local efficiency between our four treatment conditions using repeated measures one-way analysis of variance. Since we performed tests on multiple nodes, we controlled false positives using FDR correction for the number of nodes examined. When a significant effect was found, we explored this effect further using paired t-tests for each pair of our four treatment conditions, correcting for multiple testing using FDR. We present the results of this post-hoc investigation in this table. N. S. – not significant, p>0.05, after FDR correction for the number of post-hoc tests performed. We report the p-values after FDR correction.

Node	F<br>P FDR-corrected	Post-hoc	Spray vs Placebo	Spray vs Intravenous	Intravenous vs Placebo	Nebuliser vs Placebo	Nebuliser vs Intravenous	Nebuliser vs Spray
FP r (Frontal Pole Right)	10.019<br>0.024	T<br>P FDR-corrected	1.809<br>N.S.	-0.337<br>N.S.	3.449<br>0.004	1.515<br>N.S.	-0.449<br>N.S.	-0.125<br>N.S.
SFG r (Superior Frontal Gyrus Right)	10.556<br>0.010	T<br>P FDR-corrected	3.296<br>0.005	0.370<br>N.S.	2.989<br>0.009	1.549<br>N.S.	-1.304<br>N.S.	-1.523<br>N.S.
pSTG r (Superior Temporal Gyrus, posterior division Right)	8.666<br>0.047	T<br>P FDR-corrected	2.214<br>0.042	0.841<br>N.S.	1.607<br>N.S.	2.069<br>N.S.	0.854<br>N.S.	-0.229<br>N.S.
aMTG r (Middle Temporal Gyrus, anterior division Right)	9.969<br>0.034	T<br>P FDR-corrected	2.441<br>0.026	2.021<br>N.S.	0.273<br>N.S.	1.146<br>N.S.	0.725<br>N.S.	-1.326<br>N.S.
pMTG r (Middle Temporal Gyrus, posterior division Right)	9.962<br>0.035	T<br>P FDR-corrected	2.389<br>0.030	2.220<br>0.042	0.751<br>N.S.	1.140<br>N.S.	0.187<br>N.S.	-2.41<br>0.029
pSMG r (Supramarginal Gyrus, posterior division Right)	9.985<br>0.033	T<br>P FDR-corrected	-0.579<br>N.S.	1.364<br>N.S.	2.476<br>0.026	0.225<br>N.S.	0..273<br>N.S.	0.579<br>N.S.
pSMG l (Supramarginal Gyrus, posterior division Left)	8.699<br>0.041	T<br>P FDR-corrected	0.568<br>N.S.	1.008<br>N.S.	-1.499<br>N.S.	2.482<br>0.029	0.914<br>N.S.	0.568<br>N.S.
AC (Cingulate Gyrus, anterior division)	10.005<br>0.028	T<br>P FDR-corrected	1.152<br>N.S.	-1.548<br>N.S.	2.204<br>0.044	-0.144<br>N.S.	3.237<br>0.006	-0.928<br>N.S.
Cuneal r (Cuneal Cortex Right)	10.023<br>0.022	T<br>P FDR-corrected	2.246<br>0.040	1.415<br>N.S.	1.829<br>N.S.	2.797<br>0.014	1.008<br>N.S.	0.289<br>N.S.
Cuneal l (Cuneal Cortex Left)	8.681<br>0.043	T<br>P FDR-corrected	1.643<br>N.S.	0.223<br>N.S.	2.259<br>0.039	1.492<br>N.S.	-0.156<br>N.S.	-0.275<br>N.S.
Brain-Stem	10.033<br>0.021	T<br>P FDR-corrected	1.303<br>N.S.	-1.178<br>N.S.	2.677<br>0.017	0.847<br>N.S.	-1.302<br>N.S.	-0.222<br>N.S.
Cereb1 l (Cerebelum Crus1 Left)	8.694<br>0.041	T<br>P FDR-corrected	2.345<br>0.033	2.053<br>N.S.	0.873<br>N.S.	1.241<br>N.S.	0.527<br>N.S.	-1.679<br>N.S.

Supplementary Table S3 - Effects of exogenous oxytocin on node degree. For each node, we first compared degree between our four treatment conditions using repeated measures one-way analysis of variance. Since we performed tests on multiple nodes, we controlled false positives using FDR correction for the number of nodes examined. When a significant effect was found, we explored this effect further using paired t-tests for each pair of our four treatment conditions, correcting for multiple testing using FDR. We present the results of this post-hoc investigation in this table. N. S. – not significant, p>0.05, after FDR correction for the number of post-hoc tests performed. We report the p-values after FDR correction.

Node	F<br>P FDR-corrected	Post-hoc	Spray vs Placebo	Spray vs Intravenous	Intravenous vs Placebo	Nebuliser vs Placebo	Nebuliser vs Intravenous	Nebuliser vs Spray
TP r (Temporal Pole Right)	9.988<br>0.033	T<br>P FDR-corrected	-0.177<br>N.S.	1.200<br>N.S.	-2.171<br>0.046	0.435<br>N.S.	2.444<br>0.0274	0.486<br>N.S.
pSTG l (Superior Temporal Gyrus, posterior division Left)	10.001<br>0.024	T<br>P FDR-corrected	1.726<br>N.S.	1.487<br>N.S.	0.306<br>N.S.	2.758<br>0.015	2.739<br>0.015	0.771<br>N.S.
pITG r (Inferior Temporal Gyrus, posterior division Right)	7.301<br>0.049	T<br>P FDR-corrected	-2.163<br>0.047	-0.258<br>N.S.	-1.971<br>N.S.	-1.602<br>N.S.	0.946<br>N.S.	0.921<br>N.S.
ICC r (Intracalcarine Cortex Right)	9.854<br>0.037	T<br>P FDR-corrected	2.722<br>0.016	-0.467<br>N.S.	1.876<br>N.S.	-0.319<br>N.S.	-2.172<br>0.046	-2.123<br>N.S.
Precuneous	10.001<br>0.026	T<br>P FDR-corrected	-0.918<br>N.S.	1.969<br>N.S.	-2.676<br>0.017	-2.030<br>N.S.	0.401<br>N.S.	-1.050<br>N.S.
Brain-Stem	10.495<br>0.019	T<br>P FDR-corrected	2.314<br>0.035	0.753<br>N.S.	1.091<br>N.S.	-1.355<br>N.S.	-1.723<br>N.S.	-3.081<br>0.008
Cereb1 l (Cerebelum Crus1 Left)	10.137<br>0.021	T<br>P FDR-corrected	3.563<br>0.003	1.108<br>N.S.	1.679<br>N.S.	1.153<br>N.S.	-0.214<br>N.S.	-1.309<br>N.S.
Cereb1 r (Cerebelum Crus1 Right)	10.002<br>0.032	T<br>P FDR-corrected	2.603<br>0.019	0.806<br>N.S.	1.511<br>N.S.	-0.206<br>N.S.	-1.229<br>N.S.	-2.229<br>0.041
Cereb7 l (Cerebelum 7b Left)	9.974<br>0.035	T<br>P FDR-corrected	1.971<br>N.S.	1.211<br>N.S.	0.755<br>N.S.	2.745<br>0.015	1.587<br>N.S.	0.067<br>N.S.

Supplementary Figure S2 – Summary of the graph-theory metrics used in our study. In this diagram, we summarize the concepts of the four graph-theory metrics we used in the current study to investigate the effects of exogenous oxytocin on the functional connectome.

Measure	Level	Details	Schematic example
**Node degree**	Local	Number of edges of a node	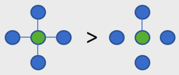
**Betweenness-centrality**	Local	Proportion of times that a node is part of an optimal minimum-distance path between any two pairs of nodes within the graph	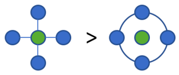
**Global Efficiency**	Global	Average of inverse-distances between each node and all other nodes in the graph; global efficiency represents a measure of graph inter-connectedness	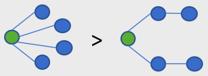
**Local Efficiency**	Local	Global efficiency of neighboring sub-graph. Local efficiency represents a measure of local integration, characterizing the degree of inter-connectedness among all nodes within a neighboring sub-graph.	

Supplementary Figure S3 – Effects of exogenous oxytocin on head motion. We compared mean frame-wise displacement between our four treatment conditions using repeated measures one-way analysis of variance. In the upper panel, we present box and violin plots depicting the distributions of mean frame-wise displacement (FD) for each treatment condition; middle horizontal lines represent the mean; boxes indicate the 25th and 75th percentiles (n=16 per treatment condition). In the lower panel, we present the descriptive statistics for each treatment condition.

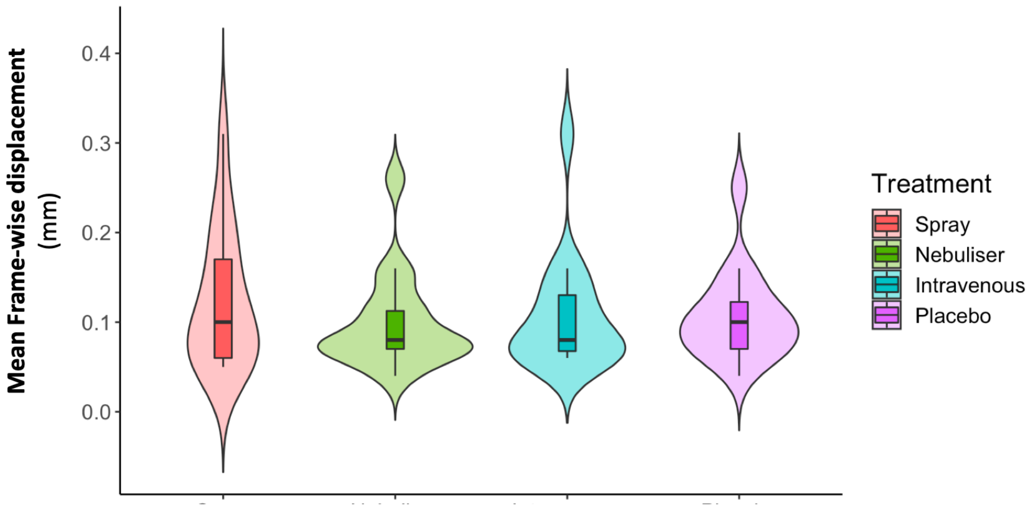

Treatment	Mean Frame-wise displacement (mm)	Standard error of the mean
**Spray**	0.123	0.019
**Nebuliser**	0.099	0.013
**Intravenous**	0.107	0.016
**Placebo**	0.106	0.013

There were no significant differences across the four treatment conditions in terms of mean FD ($F(2.521, 37.81) = 1.010$, $p = 0.389$).
