# Supplementary material for: Oxytocin modulates local topography of human functional connectome in healthy men at rest
Source: Commun Biol. 2021 Jan 15;4:68. doi: 10.1038/s42003-020-01610-z (PMC7811009; doi:10.1038/s42003-020-01610-z)
Supplement: Supplementary file 3 — Description of Additional Supplementary Files [file 42003_2020_1610_MOESM3_ESM.pdf]

Description of Additional Supplementary Files

File Name: Supplementary Data 1
Description: Source data underlying Figs. 1 and 7

File Name: Supplementary Data 2
Description: Source data underlying Supplementary Figures 1 and 3
