# Supplementary material for: Oxytocin modulates local topography of human functional connectome in healthy men at rest
Source: Commun Biol. 2021 Jan 15;4:68. doi: 10.1038/s42003-020-01610-z (PMC7811009; doi:10.1038/s42003-020-01610-z)
Supplement: Supplementary file 6 — Reporting Summary [file 42003_2020_1610_MOESM6_ESM.pdf]

nature research

Corresponding author(s): Yannis Paloyelis

Last updated by author(s): Nov 26, 2020

Reporting Summary

Nature Research wishes to improve the reproducibility of the work that we publish. This form provides structure for consistency and transparency in reporting. For further information on Nature Research policies, see our Editorial Policies and the Editorial Policy Checklist.

Statistics

For all statistical analyses, confirm that the following items are present in the figure legend, table legend, main text, or Methods section.

n/a	Confirmed	
☐	☒	The exact sample size (*n*) for each experimental group/condition, given as a discrete number and unit of measurement
☐	☒	A statement on whether measurements were taken from distinct samples or whether the same sample was measured repeatedly
☐	☒	The statistical test(s) used AND whether they are one- or two-sided<br>*Only common tests should be described solely by name; describe more complex techniques in the Methods section.*
☒	☐	A description of all covariates tested
☐	☒	A description of any assumptions or corrections, such as tests of normality and adjustment for multiple comparisons
☐	☒	A full description of the statistical parameters including central tendency (e.g. means) or other basic estimates (e.g. regression coefficient) AND variation (e.g. standard deviation) or associated estimates of uncertainty (e.g. confidence intervals)
☐	☒	For null hypothesis testing, the test statistic (e.g. F, t, r) with confidence intervals, effect sizes, degrees of freedom and P value noted<br>*Give P values as exact values whenever suitable.*
☒	☐	For Bayesian analysis, information on the choice of priors and Markov chain Monte Carlo settings
☒	☐	For hierarchical and complex designs, identification of the appropriate level for tests and full reporting of outcomes
☐	☒	Estimates of effect sizes (e.g. Cohen's *d*, Pearson's *r*), indicating how they were calculated

Our web collection on statistics for biologists contains articles on many of the points above.

Software and code

Policy information about availability of computer code

Data collection	No software was used
Data analysis	AFNI, FSL, CONN toolbox, Brain Connectivity toolbox, Matlab, SPSS 24, BrainNET viewer, NeuroSynth, ggplot from R

For manuscripts utilizing custom algorithms or software that are central to the research but not yet described in published literature, software must be made available to editors and reviewers. We strongly encourage code deposition in a community repository (e.g. GitHub). See the Nature Research guidelines for submitting code & software for further information.

Data

Policy information about availability of data

All manuscripts must include a data availability statement. This statement should provide the following information, where applicable:

- Accession codes, unique identifiers, or web links for publicly available datasets
- A list of figures that have associated raw data
- A description of any restrictions on data availability

Data can be accessed from the corresponding author upon reasonable request; raw data associated with figures is available as source data

Field-specific reporting

Life sciences study design

All studies must disclose on these points even when the disclosure is negative.

Sample size	We enrolled 17 healthy male adult volunteers (mean age 24.5, SD = 5, range 19-34 years). Our sample size would allow us to detect a medium effect size f = 0.25 with 80% of statistical power in a repeated measures one-way ANOVA testing for differences across our four treatment conditions in this study.
Data exclusions	One participant did not complete one of the four visits and for this reason was excluded from all analyses.
Replication	The study did not include a replication sample
Randomization	We employed a double-blind, placebo-controlled, triple-dummy, crossover design. Participants were randomly allocated to a treatment order (i.e. a specific plan regarding which route delivered the active drug in each experimental session) that was determined using a Latin square design.
Blinding	Data collection was double-blind.

Reporting for specific materials, systems and methods

We require information from authors about some types of materials, experimental systems and methods used in many studies. Here, indicate whether each material, system or method listed is relevant to your study. If you are not sure if a list item applies to your research, read the appropriate section before selecting a response.

Materials & experimental systems

n/a	Involved in the study	
☐	☐	Antibodies
☐	☐	Eukaryotic cell lines
☐	☐	Palaeontology and archaeology
☐	☐	Animals and other organisms
☐	☒	Human research participants
☐	☐	Clinical data
☐	☐	Dual use research of concern

Methods

n/a	Involved in the study	
☐	☐	ChIP-seq
☐	☐	Flow cytometry
☐	☒	MRI-based neuroimaging

Antibodies

Antibodies used	*Describe all antibodies used in the study; as applicable, provide supplier name, catalog number, clone name, and lot number.*
Validation	*Describe the validation of each primary antibody for the species and application, noting any validation statements on the manufacturer's website, relevant citations, antibody profiles in online databases, or data provided in the manuscript.*

Eukaryotic cell lines

Policy information about cell lines

Cell line source(s)	*State the source of each cell line used.*
Authentication	*Describe the authentication procedures for each cell line used OR declare that none of the cell lines used were authenticated.*
Mycoplasma contamination	*Confirm that all cell lines tested negative for mycoplasma contamination OR describe the results of the testing for mycoplasma contamination OR declare that the cell lines were not tested for mycoplasma contamination.*
Commonly misidentified lines (See ICLAC register)	*Name any commonly misidentified cell lines used in the study and provide a rationale for their use.*

Palaeontology and Archaeology

Specimen provenance	*Provide provenance information for specimens and describe permits that were obtained for the work (including the name of the issuing authority, the date of issue, and any identifying information).*
Specimen deposition	*Indicate where the specimens have been deposited to permit free access by other researchers.*

Dating methods	*If new dates are provided, describe how they were obtained (e.g. collection, storage, sample pretreatment and measurement), where they were obtained (i.e. lab name), the calibration program and the protocol for quality assurance OR state that no new dates are provided.*

☐ Tick this box to confirm that the raw and calibrated dates are available in the paper or in Supplementary Information.

Ethics oversight	*Identify the organization(s) that approved or provided guidance on the study protocol, OR state that no ethical approval or guidance was required and explain why not.*

Note that full information on the approval of the study protocol must also be provided in the manuscript.

Animals and other organisms

Policy information about studies involving animals; ARRIVE guidelines recommended for reporting animal research

Laboratory animals	*For laboratory animals, report species, strain, sex and age OR state that the study did not involve laboratory animals.*
Wild animals	*Provide details on animals observed in or captured in the field; report species, sex and age where possible. Describe how animals were caught and transported and what happened to captive animals after the study (if killed, explain why and describe method; if released, say where and when) OR state that the study did not involve wild animals.*
Field-collected samples	*For laboratory work with field-collected samples, describe all relevant parameters such as housing, maintenance, temperature, photoperiod and end-of-experiment protocol OR state that the study did not involve samples collected from the field.*
Ethics oversight	*Identify the organization(s) that approved or provided guidance on the study protocol, OR state that no ethical approval or guidance was required and explain why not.*

Note that full information on the approval of the study protocol must also be provided in the manuscript.

Human research participants

Policy information about studies involving human research participants

Population characteristics	We enrolled 17 healthy male adult volunteers (mean age 24.5, SD = 5, range 19-34 years). One participant did not complete one of the four visits and for this reason was excluded from all analyses. We screened participants for psychiatric conditions using the Symptom Checklist-90-Revised(72) and the Beck Depression Inventory-II(73) questionnaires. Participants were not taking any prescribed drugs, did not have a history of drug abuse and tested negative on a urine panel screening test for recreational drugs; they consumed <28 units of alcohol per week and <5 cigarettes per day. We instructed participants to abstain from alcohol and heavy exercise for 24 hours and from any beverage or food consumption for 2 hours before scanning.
Recruitment	Participants were recruited using the KCL online recruitment platform
Ethics oversight	King's College London Research Ethics Committee (PNM/13/14-163) approved the study.

Note that full information on the approval of the study protocol must also be provided in the manuscript.

Clinical data

Policy information about clinical studies

All manuscripts should comply with the ICMJEguidelines for publication of clinical research and a completedCONSORT checklist must be included with all submissions.

Clinical trial registration	*Provide the trial registration number from ClinicalTrials.gov or an equivalent agency.*
Study protocol	*Note where the full trial protocol can be accessed OR if not available, explain why.*
Data collection	*Describe the settings and locales of data collection, noting the time periods of recruitment and data collection.*
Outcomes	*Describe how you pre-defined primary and secondary outcome measures and how you assessed these measures.*

Dual use research of concern

Policy information about dual use research of concern

Hazards

Could the accidental, deliberate or reckless misuse of agents or technologies generated in the work, or the application of information presented in the manuscript, pose a threat to:

No | Yes

- [ ] [ ] Public health
- [ ] [ ] National security
- [ ] [ ] Crops and/or livestock
- [ ] [ ] Ecosystems
- [ ] [ ] Any other significant area

Experiments of concern

Does the work involve any of these experiments of concern:

No | Yes

- [ ] [ ] Demonstrate how to render a vaccine ineffective
- [ ] [ ] Confer resistance to therapeutically useful antibiotics or antiviral agents
- [ ] [ ] Enhance the virulence of a pathogen or render a nonpathogen virulent
- [ ] [ ] Increase transmissibility of a pathogen
- [ ] [ ] Alter the host range of a pathogen
- [ ] [ ] Enable evasion of diagnostic/detection modalities
- [ ] [ ] Enable the weaponization of a biological agent or toxin
- [ ] [ ] Any other potentially harmful combination of experiments and agents

ChIP-seq

Data deposition

- [ ] Confirm that both raw and final processed data have been deposited in a public database such as GEO.
- [ ] Confirm that you have deposited or provided access to graph files (e.g. BED files) for the called peaks.

Data access links
May remain private before publication.
For "Initial submission" or "Revised version" documents, provide reviewer access links. For your "Final submission" document, provide a link to the deposited data.

Files in database submission
Provide a list of all files available in the database submission.

Genome browser session
(e.g. UCSC)
Provide a link to an anonymized genome browser session for "Initial submission" and "Revised version" documents only, to enable peer review. Write "no longer applicable" for "Final submission" documents.

Methodology

Replicates
Describe the experimental replicates, specifying number, type and replicate agreement.

Sequencing depth
Describe the sequencing depth for each experiment, providing the total number of reads, uniquely mapped reads, length of reads and whether they were paired- or single-end.

Antibodies
Describe the antibodies used for the ChIP-seq experiments; as applicable, provide supplier name, catalog number, clone name, and lot number.

Peak calling parameters
Specify the command line program and parameters used for read mapping and peak calling, including the ChIP, control and index files used.

Data quality
Describe the methods used to ensure data quality in full detail, including how many peaks are at FDR 5% and above 5-fold enrichment.

Software
Describe the software used to collect and analyze the ChIP-seq data. For custom code that has been deposited into a community repository, provide accession details.

Flow Cytometry

Plots

Confirm that:

☐ The axis labels state the marker and fluorochrome used (e.g. CD4-FITC).

☐ The axis scales are clearly visible. Include numbers along axes only for bottom left plot of group (a 'group' is an analysis of identical markers).

☐ All plots are contour plots with outliers or pseudocolor plots.

☐ A numerical value for number of cells or percentage (with statistics) is provided.

Methodology

Sample preparation	*Describe the sample preparation, detailing the biological source of the cells and any tissue processing steps used.*
Instrument	*Identify the instrument used for data collection, specifying make and model number.*
Software	*Describe the software used to collect and analyze the flow cytometry data. For custom code that has been deposited into a community repository, provide accession details.*
Cell population abundance	*Describe the abundance of the relevant cell populations within post-sort fractions, providing details on the purity of the samples and how it was determined.*
Gating strategy	*Describe the gating strategy used for all relevant experiments, specifying the preliminary FSC/SSC gates of the starting cell population, indicating where boundaries between "positive" and "negative" staining cell populations are defined.*

☐ Tick this box to confirm that a figure exemplifying the gating strategy is provided in the Supplementary Information.

Magnetic resonance imaging

Experimental design

Design type	Resting-state
Design specifications	NA
Behavioral performance measures	NA

Acquisition

Imaging type(s)	BOLD fMRI
Field strength	3T
Sequence & imaging parameters	MRI data were acquired on a 3T MR750 system (GE Healthcare, Waukesha, Wisconsin) with a 32-channel head coil. Functional data was acquired using a shot multi-echo echo planar imaging (EPI) sequence (TR = 2500 ms, TE’s= 12, 28, 44 and 60 ms, FA= 80°, FOV= 240 mm2, Matrix= 64x 64, Slice thickness= 3 mm, 32 continuous descending axial slices). Multi-echo fMRI data acquisition has been shown to optimize sensitivity to detect BOLD signal over noise sources(80). The resting state fMRI scan lasted 8 min 10 s, with collection of a total of 192 volumes for each echo. A 3D high-spatial-resolution, Magnetisation Prepared Rapid Acquisition (3D MPRAGE) T1-weighted scan was acquired (FOV = 270 mm2 TR/TE/TI = 7.328/3.024/400 ms, resolution = 1.1 x 1.1 x 1.2 mm).
Area of acquisition	Whole-brain

Diffusion MRI ☐ Used ☒ Not used

Preprocessing

Preprocessing software	AFNI, FSL, CONN toolbox, Brain Connectivity toolbox
Normalization	We built a study-specific template representing the average T1-weighted anatomical image using the Advanced Normalization Tools (ANTs) toolbox (89). Finally, each participant's cleaned datasets were co-registered to their corresponding structural scan, normalized to the study-specific template before warping to standard MNI152 space, with 2 mm3 resampling. The final normalised images were visually inspected to ensure the quality of the pre-processing and the absence of artefacts.
Normalization template	Standard MNI152 space
Noise and artifact removal	The Multi-Echo ICA approach included in the meica.py tool was used to remove from the OC data motion artefacts and other

sources of noise; this approach has shown greater efficacy in reducing non-BOLD sources of noise and increasing the temporal signal-to-noise ratio when compared to other de-noising approaches.

Volume censoring: Not implemented

Statistical modeling & inference

Model type and settings: Univariate analysis

Effect(s) tested: Repeated-measures ONE-way ANOVA and paired T-tests

Specify type of analysis: ☐ Whole brain ☒ ROI-based ☐ Both

Anatomical location(s): 132 non-overlapping ROIs spanning cortical and subcortical areas and the cerebellum using a combination of the cortical and subcortical areas from the FSL Harvard-Oxford atlas and of the cerebellar areas of the Automatic Anatomical Labelling atlas

Statistic type for inference (See Eklund et al. 2016): NA

Correction: FDR correction for number of ROIs and treatment groups

Models & analysis

n/a	Involved in the study
☐	☐ Functional and/or effective connectivity
☐	☒ Graph analysis
☐	☐ Multivariate modeling or predictive analysis

Functional and/or effective connectivity: Pearson correlation

Graph analysis: We used an undirected signed weighted approach in which we kept information about the sign and weight of each of the connections between the nodes (ROIs) of our network, without making assumptions about the direction of this relationship. We computed four global and regional network metrics: global efficiency, node degree, local efficiency and betweenness-centrality. We used the fully weighted networks to calculate equi-sparse networks by retaining a fixed percentage (K) of the total amount of possible edges. In order to determine systematic pharmacological effects on the network's topological organization that are not dependent on the choice of an arbitrary threshold, we chose a range of sparsity thresholds from 5 to 34%, with steps of 1%, based on previous evidence showing the characteristic small-world behaviour of human brain networks is most consistently observed for this range. For each of the graph metrics analysed, we summarized the different values over the range of thresholds using the area under the curve (AUC), instead of applying multiple testing for each metric/threshold.

Multivariate modeling and predictive analysis: *Specify independent variables, features extraction and dimension reduction, model, training and evaluation metrics.*
